# The Clinical Significance of the Early Screening of Keratoconus and Its Impact on Maintaining Quality of Life

**DOI:** 10.3390/life16010124

**Published:** 2026-01-14

**Authors:** Mimoza Ismaili

**Affiliations:** Department of Ophthalmology, Faculty of Medicine, University of Pristina “Hasan Pristina”, 10000 Pristina, Kosovo; drmimozaismaili@gmail.com; Tel.: +383-44-2660-12

**Keywords:** keratoconus, corneal thickness, corneal topography

## Abstract

Background: This study focuses on the diagnosis and treatment of keratoconus in the early stage and aims to identify the environmental and risk factors that contribute to its progression. Methods: This retrospective investigation was carried out at the University Clinical Center of Kosovo (UCCK) and comprised 131 patients newly diagnosed with keratoconus (KC). All procedures adhered to the Declaration of Helsinki, and the University of Pristina ethics committee approved this study before its initiation (Ref.Nr.104/2023). The confidentiality and anonymity of the surveyed patients were respected. The patients’ data consisted of gender, age, and race. Results: There were significant differences in the K1 distribution between groups, as the normal group (41.4 ± 0.5) was significantly lower than the suspect group (45.0 ± 3.2) and the degree of keratoconus (*p* < 0.001). There were significant differences in K2 between the groups, as the normal group (44.7 ± 5.1) was significantly lower than the suspect group (47.1 ± 2.8) and the other grades of keratoconus (*p* < 0.001). There were significant differences between groups regarding Kmax, as the normal group (44.5 ± 3.1) was significantly lower than the suspect group (46.9 ± 1.6) and the other grades of keratoconus (where *p* < 0.001). Statistically meaningful differences were detected between the groups with respect to subtlety, as the normal group (504.0 ± 27.6) was significantly higher than the suspect group (499.0 ± 48.1) and the other degrees of keratoconus (*p* < 0.001). Conclusions: Disease progression can significantly affect vision; therefore, early screening enables timely treatment (CXL). The evolution of this technique has contributed to preventing and slowing disease progression.

## 1. Introduction

Keratoconus (KC) is a progressive ectatic corneal disorder characterized by increasing corneal curvature and thinning, typically manifesting during adolescence and rarely progressing beyond the fourth decade of life [1,2,3]. The disease is marked by progressive stromal thinning and biomechanical weakening, changes that often precede clinically detectable topographic abnormalities [3]. Keratoconus frequently begins unilaterally but commonly progresses to bilateral involvement, resulting in the development of myopia and irregular astigmatism [4,5]. These refractive changes lead to reduced visual acuity (VA), increased light sensitivity, monocular diplopia, and visual distortion, all of which may significantly impair daily functioning [1,6].

The etiology of keratoconus is multifactorial, involving both genetic predisposition and environmental influences, including eye rubbing associated with allergic and dry eye conditions [7,8,9]. While advanced stages of the disease are associated with marked visual deterioration and reduced best-corrected visual acuity (BCVA), early keratoconus may present with minimal symptoms and preserved visual acuity, often resembling simple refractive error [1]. This asymptomatic or subclinical phase poses a diagnostic challenge but represents a critical window for intervention aimed at halting disease progression.

Recent advances in anterior segment imaging, particularly corneal tomography (CT), have significantly enhanced the ability to detect keratoconus at its earliest stages [10]. Tomographic assessment of both anterior and posterior corneal elevation, combined with pachymetric mapping, allows for improved identification of early ectatic changes that are not evident on conventional topography [11,12]. Early diagnosis has become especially important in the context of timely therapeutic interventions, such as corneal collagen cross-linking, which can stabilize the cornea and reduce the risk of long-term visual impairment.

Beyond measurable clinical parameters, keratoconus imposes a substantial burden on patients’ quality of life. Visual distortions, reduced contrast sensitivity, fluctuating vision, and chronic visual discomfort can negatively affect educational performance, professional productivity, and social interactions, and they may contribute to psychological distress [6,11]. Visual quality of life (QoL) is commonly assessed using validated patient-reported outcome measures, such as the National Eye Institute Visual Function Questionnaire (NEI VFQ-25) and keratoconus-specific instruments, which capture the functional and psychosocial impact of visual impairment. However, in clinical practice and research, the relationship between early keratoconus detection and preservation of quality of life remains insufficiently explored, particularly in populations identified through early screening strategies.

Although the importance of early diagnosis and the diagnostic utility of corneal tomography are well established, there is a relative lack of data linking early keratoconus screening to clinically meaningful outcomes related to quality of life. Specifically, it remains unclear to what extent early identification of keratoconus, prior to significant functional decline, may contribute to maintaining visual function and minimizing long-term psychosocial consequences.

Therefore, the aim of this study is to evaluate the clinical significance of early keratoconus screening using corneal tomography and to examine its potential impact on preserving visual function and quality of life. By focusing on patients identified at an early stage of the disease, this study seeks to clarify the added value of early detection strategies in reducing disease burden and improving long-term patient-centered outcomes.

## 2. Materials and Methods

This study included 131 respondents. In total, 262 eyes were recruited from referrals to the University Clinical Center, Department of Ophthalmology. Age, gender, visual acuity (VA), and keratometry were recorded, along with clinical signs and symptoms, from all respondents, before being retrospectively evaluated. Written informed consent was obtained from the participants before initiating this study, and possible risks were explained. All procedures adhered to the Declaration of Helsinki, and the University of Pristina ethics committee approved this study before commencement (104/2023). This retrospective study included consecutive patients referred to the Department of Ophthalmology, University Clinical Center of Kosovo, during the period from January 2020 to December 2023. A total of 131 patients (262 eyes) were evaluated. Both eyes were clinically examined in all patients; however, statistical analyses were performed using a predefined eye-selection strategy to avoid inter-eye correlation. The study population included patients with unilateral or bilateral keratoconus, as well as individuals classified as keratoconus suspects based on tomographic findings. The participants had clear signs of keratoconus, including previously diagnosed and patients newly diagnosed keratoconus patients. Topographic examination was used to record keratometries of the following basic parameters: flatter, steeper, maximal keratometry, and corneal pachymometry. A diagnosis of suspected keratoconus was established when suspicious findings were present bilaterally or when unilateral suspicion coexisted with a clinically normal fellow eye. The most frequently observed clinical indicator in our cohort was habitual eye rubbing. Environmental factors, such as genetic factors, atopy, allergy, and dry eye, also affected its pathogenesis. Although keratoconus is known to exhibit significant inter-eye asymmetry, particularly in early stages, only right-eye (OD) data were included in the primary analysis to avoid statistical bias related to inter-eye correlation. In our cohort, bilateral tomographic assessment demonstrated comparable distributions of disease stage and keratometric parameters between eyes at the population level. Importantly, tomographic screening frequently revealed subtle ectatic changes in the fellow eye, even when one eye appeared clinically normal, supporting the concept of bilateral involvement with asymmetric expression rather than true unilateral disease.

In the context of this study, the term early keratoconus screening refers to the detection of both subclinical keratoconus (forme fruste) and Amsler–Krumeich grade 1 keratoconus. Subclinical keratoconus was defined as the presence of suspicious tomographic findings (including abnormal posterior elevation, pachymetric progression, or asymmetric corneal thickness distribution) in the absence of clinical signs and with preserved visual acuity. Grade 1 keratoconus was defined according to the Amsler–Krumeich classification as cases with mean keratometry values < 48 D, mild irregular astigmatism, and no corneal scarring. This diagnostic threshold was selected to reflect the earliest stages at which intervention may prevent disease progression (Keratoconus severity was classified according to the Amsler–Krumeich (AK) classification system, using mean keratometry (Km), refractive error, corneal thickness, and clinical signs. Grade 1 keratoconus was defined as Km < 48.0 D with mild irregular astigmatism and absence of corneal scarring; Grade 2 as Km 48.0–53.0 D; Grade 3 as Km 53.0–55.0 D with increasing stromal thinning; and Grade 4 as Km > 55.0 D with marked corneal thinning and scarring. These criteria were applied consistently across all analyses.

Suspected keratoconus was defined based on Scheimpflug tomographic criteria obtained using the Pentacam HR system, including abnormal posterior elevation, asymmetric pachymetric progression, and deviation in corneal thickness distribution, in the absence of slit-lamp signs. Eyes with borderline keratometric values and tomographic abnormalities not fulfilling full AK criteria were classified as keratoconus suspects, consistent with previously published Pentacam-based diagnostic approaches.

Data collection. Among the participants, 26 eyes demonstrated bilateral KC, while another 26 exhibited unilateral involvement with differing severities. Asymmetric refractive astigmatism was characteristic of mild to moderate stages, whereas progressive astigmatism predominated in advanced KC. Disease classification incorporated clinical evolution, ocular signs, and corneal topography. We used the Amsler–Krumeich (AK) classification to determine the KCN degree of severity in 4 stages: early stage (KM < 48 D), second stage (KM < 48–53 KD), third stage (KM = > 53 D), and advanced grade (KM > 55 D). Suspected KC was confirmed when suspicion was identified in both eyes or when one eye showed suspicious findings and the contralateral eye appeared normal.

Inclusion criteria: subjects with tomographic signs of KC based on Scheimpflug imaging, individuals with myopia or myopic astigmatism, participants without coexisting ocular disease, and those with no history of ocular surgery.

Exclusion criteria: glaucoma, a history of corneal refractive procedures, other anterior segment abnormalities (e.g., corneal scarring, edema), staphyloma, absence of informed consent, and severe KC.

Procedure: Over a three-year period, data from 262 eyes were retrospectively obtained. After informed consent, each respondent underwent full ophthalmic examination, including slit-lamp evaluation of the anterior segment, Fleischer’s ring, Vogt’s striae, and conical protrusion, corneal tomography with the Pentacam HR system (Oculus, Wetzlar, Germany), and retinoscopy (scissor reflex sign). Objective refraction (autorefractometry) and subjective refraction using the Snellen chart were performed. Because left-eye findings paralleled right-eye outcomes, only right-eye data are reported. To minimize bias related to within-subject inter-eye correlation, only right-eye (OD) data were included in quantitative statistical analyses. Although keratoconus is frequently asymmetric, exploratory analysis demonstrated comparable distributions of keratometric and pachymetric parameters between right and left eyes at the cohort level. Fellow-eye data were used descriptively to assess bilaterality and asymmetry but were not included in inferential statistics.

Statistical analysis: All data were analyzed using the SPSS statistical package 22.0 bn. The acquired data are presented in tables and charts. The structure of the index, the arithmetic mean, the standard deviation, and the minimal and maximal values were calculated from the statistical parameters. The qualitative data were tested using the χ^2^ test and Fisher’s test, which had a normal distribution in the T-test. The correlation between the two phenomena was performed with Spearman’s correlation. The difference is significant if *p* < 0.05. Normality of continuous variables was assessed using the Shapiro–Wilk test. Between-group comparisons were performed using one-way ANOVA for normally distributed variables, with post hoc analysis where appropriate, while non-parametric tests were applied when distributional assumptions were not met. Categorical variables were analyzed using the chi-square test or Fisher’s exact test, as appropriate. Correlations were evaluated using Spearman’s rank correlation coefficient. A *p*-value <0.05 was considered statistically significant.

Limitations: This study has several limitations. Its retrospective design precluded the use of validated vision-related or keratoconus-specific quality of life questionnaires; therefore, the impact of keratoconus and early detection on quality of life was inferred indirectly from clinical and tomographic parameters rather than measured as an independent outcome. The inclusion of patients across different stages of disease enabled stage-based comparison but may have introduced heterogeneity that limits conclusions specifically related to early screening. Only right-eye data were analyzed to avoid inter-eye correlation, which may have reduced the amount of available bilateral information. In addition, the single-center design and lack of longitudinal follow-up limit the generalizability of the findings and preclude assessment of long-term functional or patient-reported outcomes. Prospective studies incorporating validated quality of life instruments and longitudinal evaluation are warranted to better define the patient-centered benefits of early keratoconus screening.

## 3. Results

**Figure 1:**
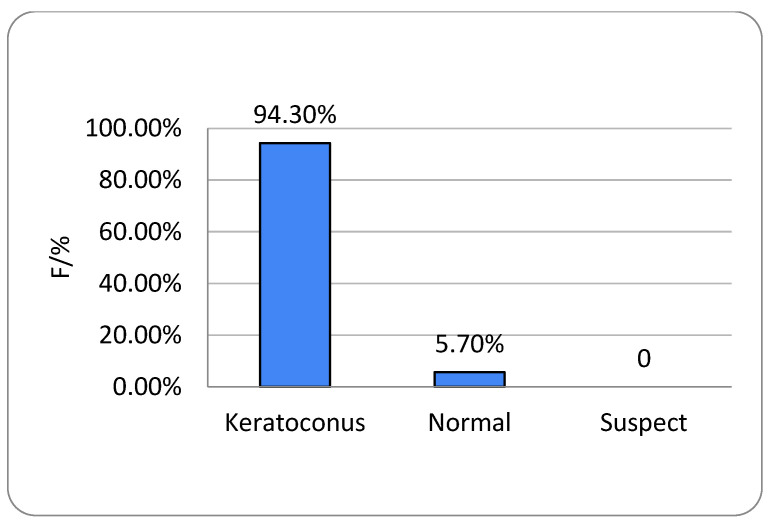
Keratoconus prevalence in females; right (OD) eyes (n = number of patients).

**Figure 2:**
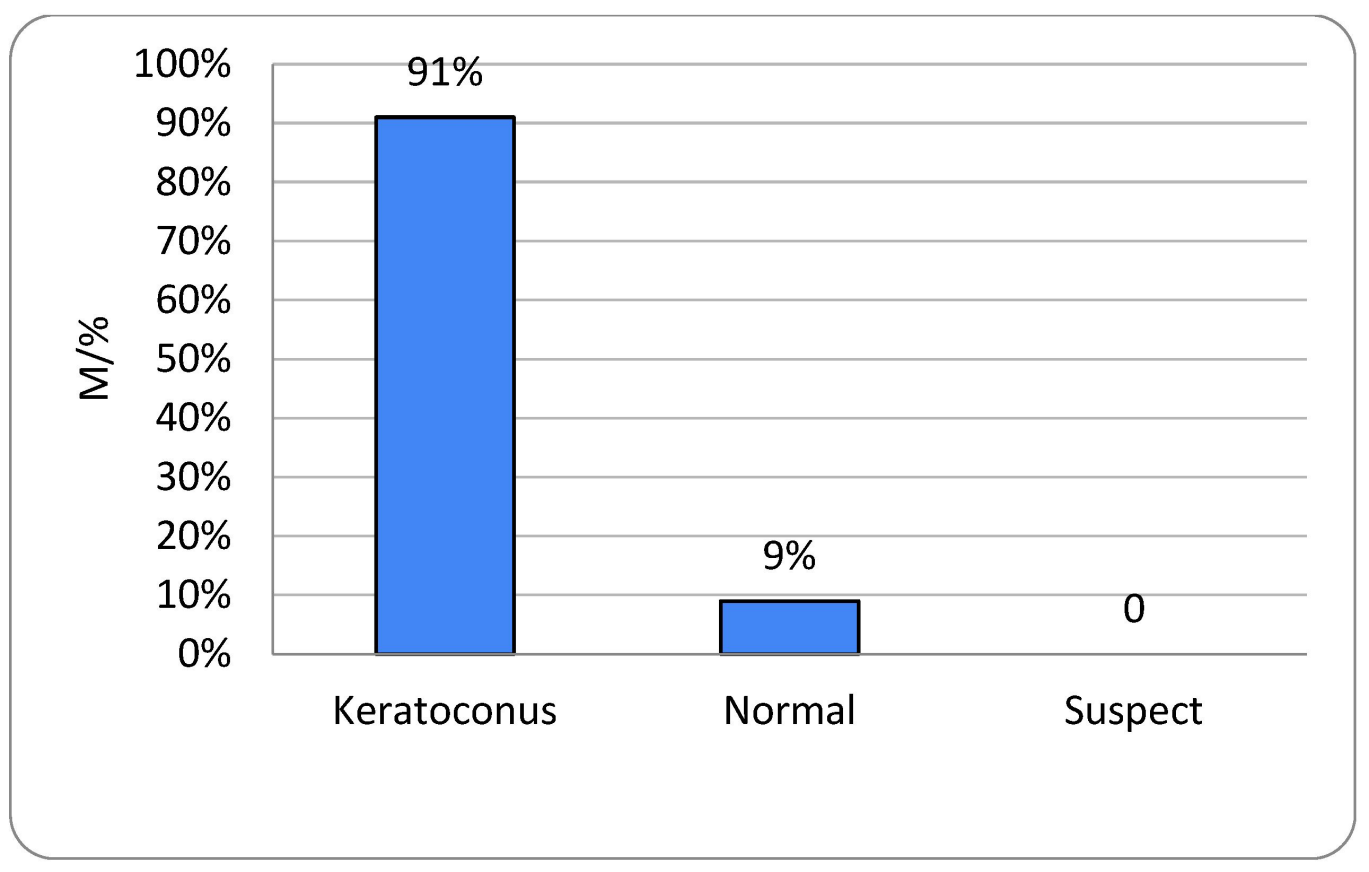
Keratoconus prevalence in men; left (OS) eyes (n = number of patients).

This study included 131 patients, of which 78 (59.5%) were men, with a mean age of 23.4 years (SD ± 4.9 years) and a range of 13 to 37 years. Of the 262 eyes examined, 160 (61.1%) received surgical treatment: 67 (41.9%) underwent the Epi-off CXL technique and 93 (58.1%) underwent the Epi-on method (Table 1; Figure 1 and Figure 2).

Among 152 individuals who reported eye rubbing, 142 (93.4%) had KC, whereas 100 of the 110 participants who denied eye rubbing (90.9%) did not have KC. A positive family history was reported in 10.7%. The relative risk of eye rubbing among KC patients was 1.174 (95% CI: 0.747–1.842), without statistical significance (*p* = 0.486). Spearman correlation showed no meaningful association between KC and eye rubbing (Table 2).

Of the 262 eyes, 240 (91.6%) were classified as KC, 2 (0.8%) as suspicious, and 20 (7.6%) as normal. More than half (55%) were categorized as grade 1 KC. KC prevalence did not differ significantly according to sex or disease grade (*p* = 0.935) (Table 3).

All participants had unilateral or bilateral KC. The right eye showed no keratoconic changes in 6.9% of subjects and the left eye in 8.4%, with no gender-related differences (*p* = 0.739 and *p* = 0.999, respectively) (Figure 1 and Figure 2). One-way ANOVA showed no significant relationship between age and either KC presence or KC severity (*p* = 0.235) (Table 1).

**Figure 3:**
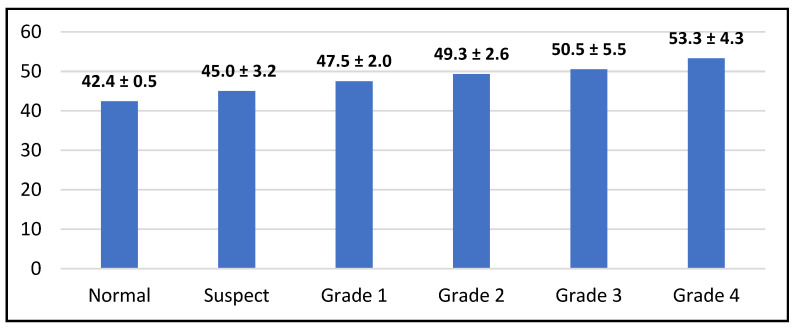
Distribution of K1 readings according to study group (n = number of patients).

**Figure 4:**
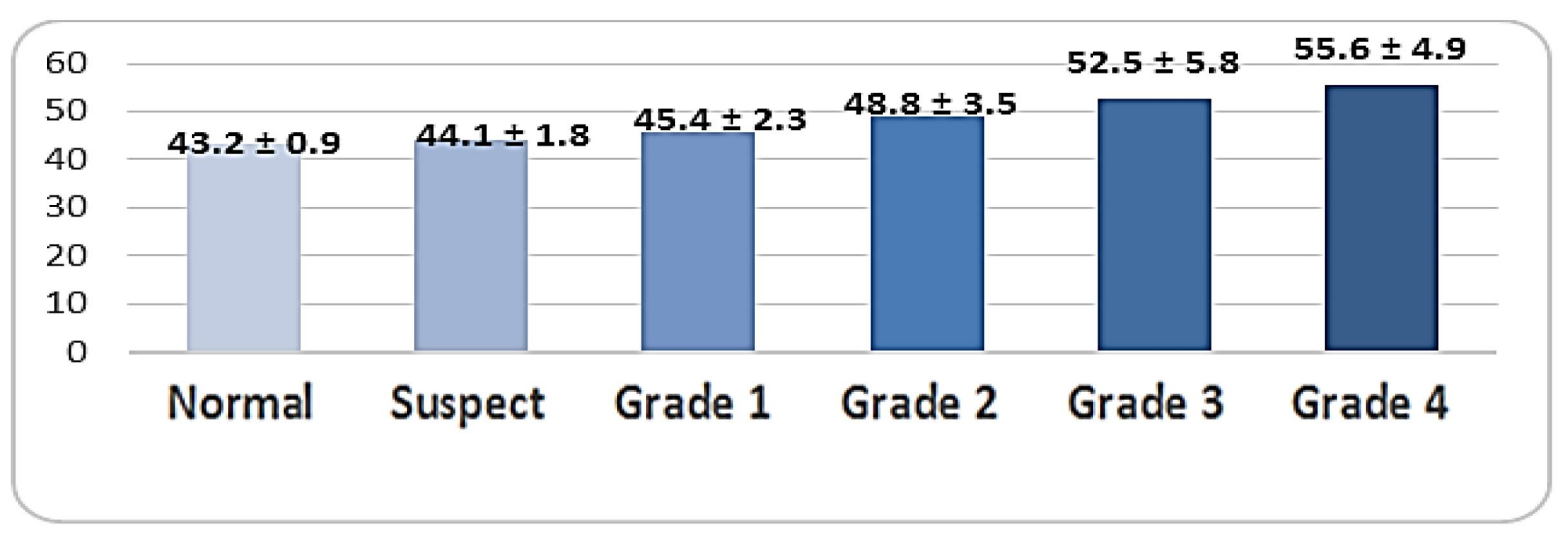
Distribution of K2 readings according to study group (n = number of patients).

**Figure 5:**
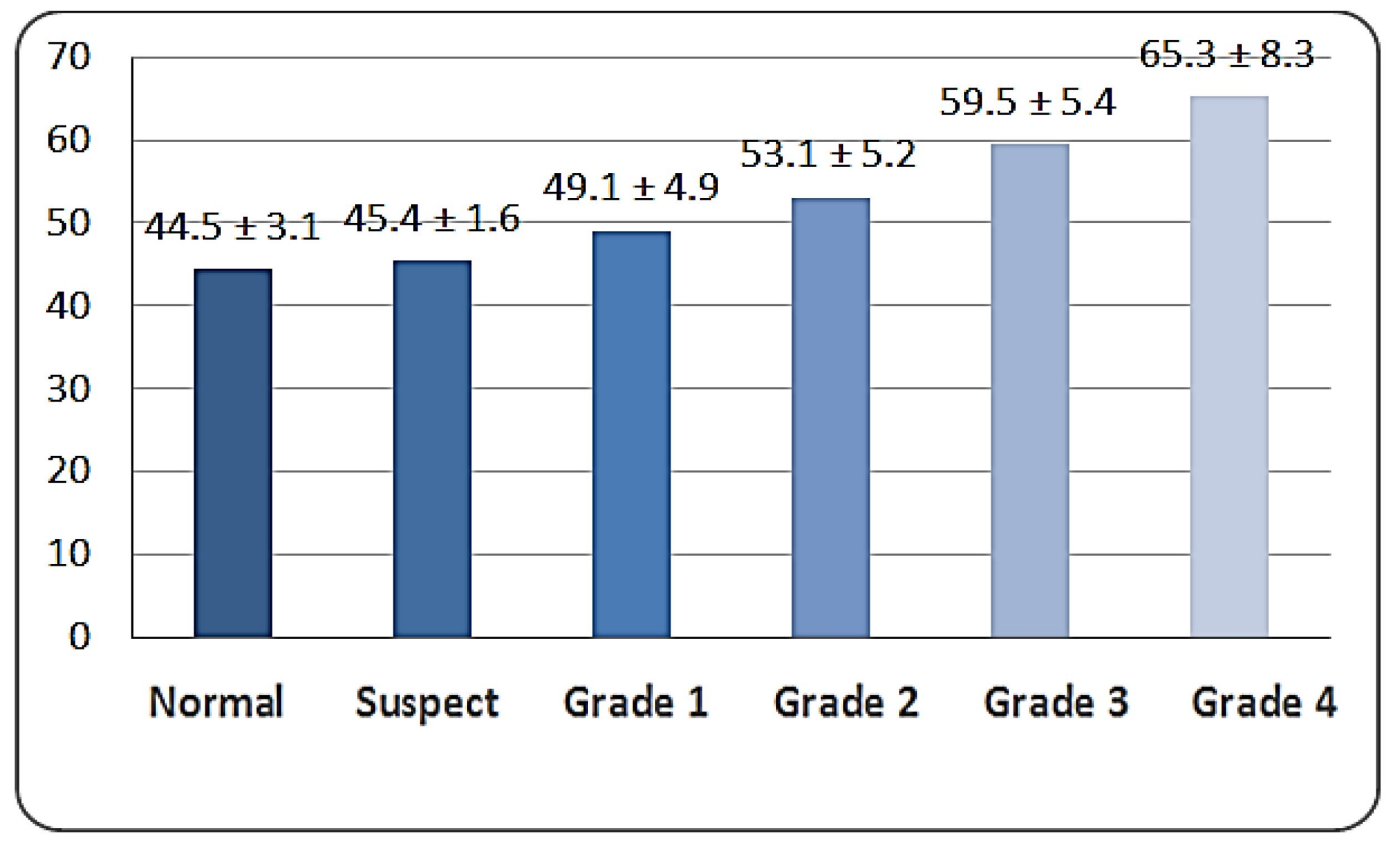
Distribution of Kmax readings according to study group (n = number of patients).

There was a significant difference in the K1 distribution among groups, as the normal group (41.4 ± 0.5) was significantly lower than the suspect group (45.0 ± 3.2) and the other grades of keratoconus (*p* < 0.001). There was a significant difference in the K2 distribution among groups, as the normal group (44.7 ± 5.1) was significantly lower than the suspect group (47.1 ± 2.8) and the other grades of keratoconus (*p* < 0.001). There was a significant difference in Kmax among groups, as the normal group (44.5 ± 3.1) was significantly lower than the suspect group (46.9 ± 1.6) and the other grades of keratoconus (*p* < 0.001) (Figure 3, Figure 4 and Figure 5).

**Figure 6:**
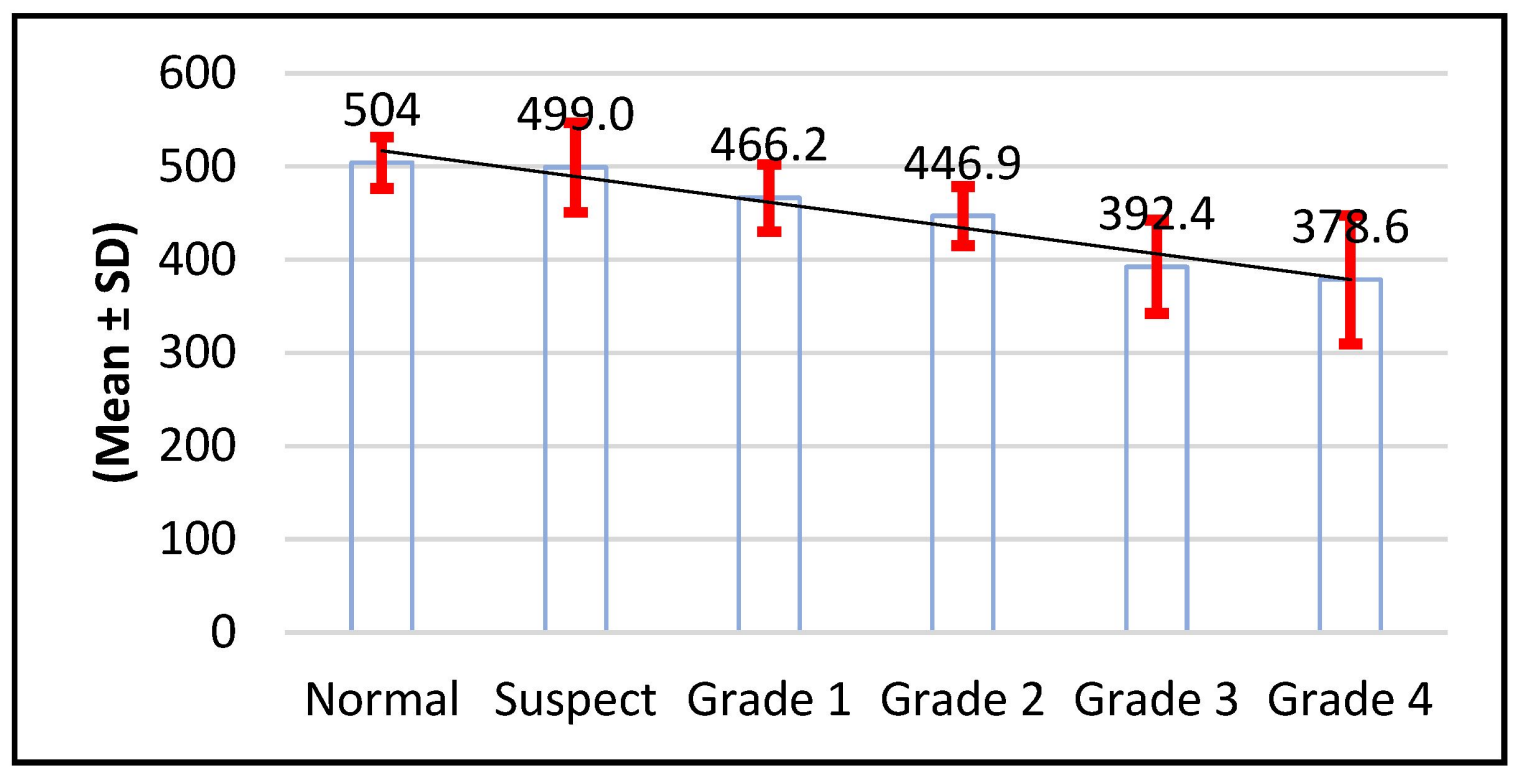
Distribution of cornea thickness values according to study group (n = number of patients).

There was a significant difference in thinness among groups, as the normal group (504.0 ± 27.6) was significantly higher than the suspect group (499.0 ± 48.1) and the other grades of keratoconus (*p* < 0.001) (Figure 6).

## 4. Discussion

**Figure 7:**
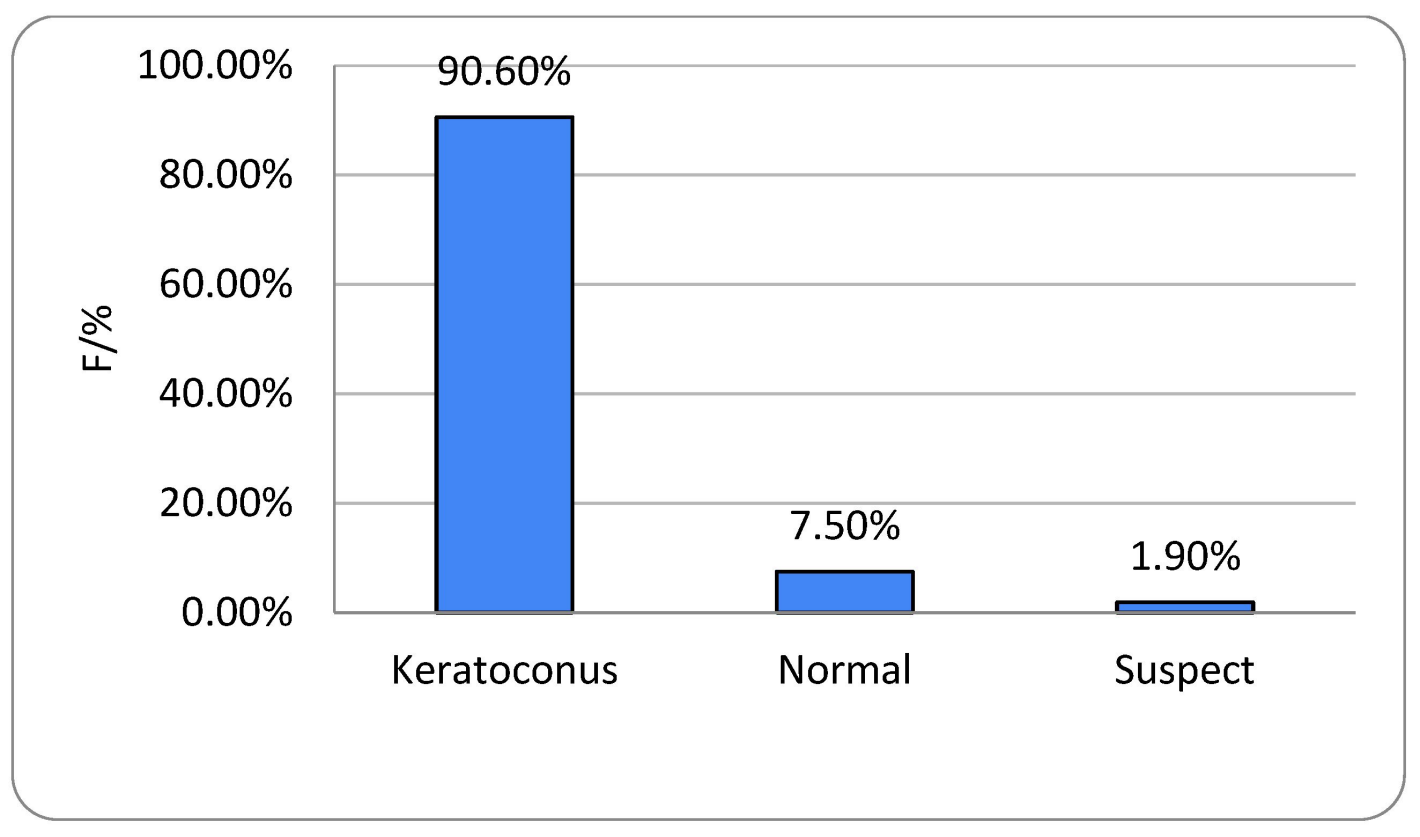
Keratoconus prevalence in females; left (OS) eyes (n = number of patients).

**Figure 8:**
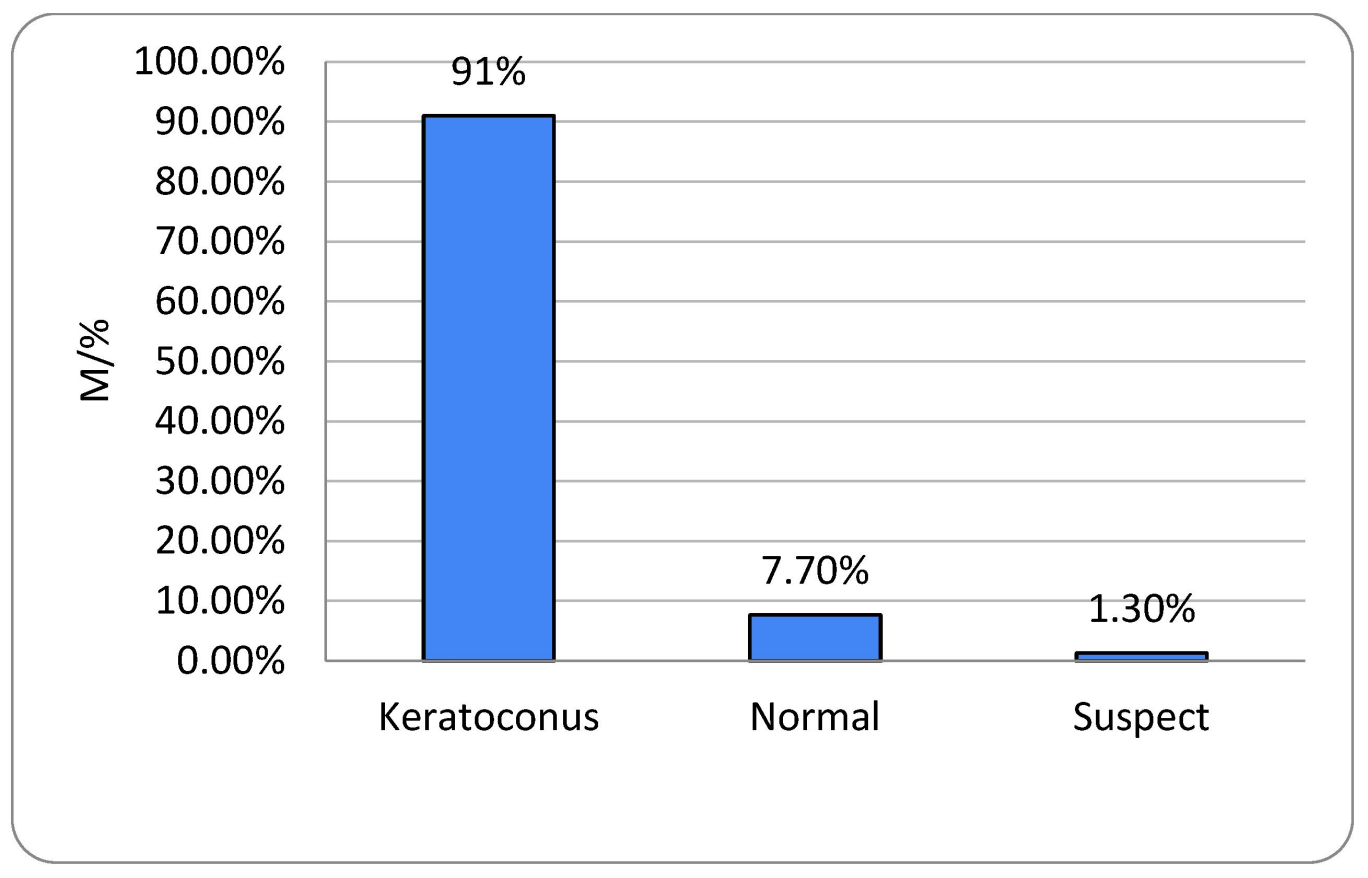
Keratoconus prevalence men; right (OD) eyes (n = number of patients).

All respondents in this research were aged 13–37 years, and the average age was 23.4 years (SD ± 4.9). The rate of KC progression varies substantially among individuals, though younger patients typically exhibit more rapid advancement [13]. KC most commonly arises around puberty and continues to progress into the third or fourth decade of life. Of the 131 respondents with KC included in our research, 78 (59.5%) were men and 53 (40.5%) were women. We can conclude that men were significantly more affected than women (Figure 1, Figure 2, Figure 7 and Figure 8). It is recognized in the literature that demographic structure can significantly influence the frequency of disease detection: earlier onset is often associated with faster progression, which may explain the predominantly young age group in our sample. Thus, we believe that gender differences in the prevalence of KC are probably not only the result of biological determinants but also of different factors, which is in accordance with the findings of other authors [3,13,14].

Multiple studies have reported higher KC prevalence among males (53–62%) [1,3,13,14,15,16,17,18,19,20], consistent with our results, though other investigations have documented female predominance (53–66%). Hashemi et al. reported an overall prevalence of 3.3% in Tehran, with 0.8% prevalence in individuals aged 14–29 and 7.5% among those older than 60 [21].

KC occurrence peaks between ages 20 and 30 and may progress until approximately age 35. Our study similarly observed progression up to age 37 (Table 1). Hashemi et al. documented increased KC prevalence in rural regions, whereas our findings indicated greater prevalence among urban residents, possibly due to increasing urban–rural overlap. Comparable prevalence patterns have been reported in northern Iran at a somewhat higher rate. Mohaghegh et al. found a 20-fold increased likelihood of KC among individuals with a positive family history, while Wang et al. estimated the risk to be 15–67 times higher [3,22,23]. We can deduce that the differences between urban and rural areas may arise from various environmental factors, the availability of health care and diagnostic capacities. The premise is that urbanization is accompanied by greater exposure to pollutants and oxidative stress, which may contribute to corneal biomechanical instability. Additionally, the strong association between a positive family history and the development of KC supports the theory of the polygenic basis of the disease [22,23].

**Figure 9:**
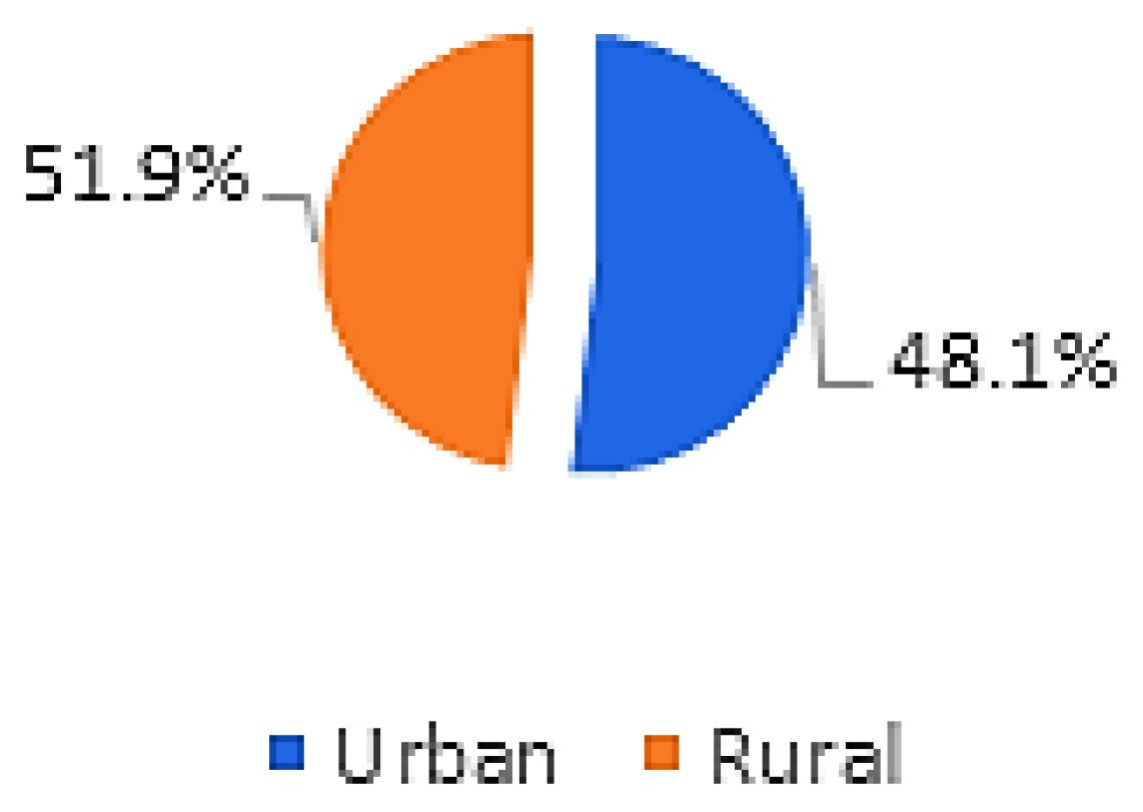
Demographic characteristics of the study participants (n = number of patients).

Eye rubbing was the most frequent environmental cause in the etiology of KC, which was observed in 93.4% of all respondents (keratoconic eyes). Although sporadic KC is predominant, 10.7% of our participants reported a positive family history, aligning with prior literature [24]. Our research findings are similar to those of other studies, which showed that eye rubbing is associated with and impacts disease progression, whether gentle friction or vigorous knuckle-grinding rubbing [25]. Our data indicated that KC patients had a 1.174-fold increased risk of reporting eye rubbing compared with other risk factors (Table 2; Figure 9). The high frequency of eye rubbing in our sample strongly supports the pathogenesis hypothesis, according to which repeated microtrauma causes keratocyte apoptosis and progressive stromal thinning [24].

Our results are similar to those of other studies, in which chronic eye rubbing was linked with keratoconus development [6]. Some studies have found associations between eye rubbing and ocular irritation, while others have attributed rubbing behavior to allergic conjunctivitis or dry-eye symptoms [3,24,26]. McGhee found that 40% of patients with KC had constant eye rubbing [27]. According to Rabinowitz’s study, 83% of 218 patients with KC had eye rubbing, similar to our study [28]. A Saudi Arabian study similarly concluded that eye rubbing frequently co-occurs with additional risk factors. Several investigations have shown that asymmetric KC is linked to more forceful rubbing, and some report KC onset approximately 14 months after chronic strong rubbing. Conversely, Owens and Gamble and Millodot et al. found no significant association (Table 2) [3,29,30,31,32]. Although causality findings are sometimes contradictory, biomechanical models very succinctly show that repeated pressure on an already predisposed cornea accelerates the loss of its strength. The observed range of findings likely reflects differences in the intensity of rubbing, the presence of inflammation, and the duration of symptoms [30,31,32]. Although a high proportion of patients with keratoconus reported habitual eye rubbing, the calculated relative risk did not reach statistical significance. This apparent discrepancy may reflect reporting bias, as eye rubbing was self-reported, as well as temporal ambiguity, since rubbing may represent both a contributing factor and a behavioral response to visual discomfort. Given the retrospective design, causal inference cannot be established, and these findings should be interpreted with caution.

KC is the leading cause of corneal ectasia, and multiple clinical trials support corneal collagen cross-linking (CXL) as an effective means of slowing or arresting disease progression. Treatment selection depends on corneal parameters, disease severity, and degree of visual impairment. The Dresden protocol recommends a minimum corneal thickness of 400 µm. Since Wollensak et al. first introduced CXL in 2003, the need for corneal transplantation has decreased [3,33,34,35,36,37,38]. Many patients still require contact lenses post-treatment, though fitting tends to be easier. In our study, both Epi-off and Epi-on CXL techniques were used for early KC to prevent further progression. Although CXL cannot reverse existing thinning, early intervention is particularly valuable given the higher risk of progression in younger individuals. Over the last decade, both CXL utilization and reported efficacy have increased significantly. Based on disease characteristics, 61.1% of our cohort underwent CXL and 38.9% were managed non-surgically [3,34,39,40,41,42]. The rise in the use of CXL therapy has been accompanied by the development of personalized protocols, including accelerated, individualized and topographically guided modalities. Although the Epi-off approach still shows the strongest biomechanical effect, new techniques tend to optimize the relationship between safety and efficiency according to the findings in [34,39,40]. The choice between Epi-off and Epi-on corneal collagen cross-linking (CXL) techniques was based on corneal thickness, patient age, disease stage, and safety considerations. The Epi-off technique was preferentially applied in eyes with sufficient corneal thickness (≥400 µm), as it allows for deeper stromal penetration of riboflavin and is associated with a stronger biomechanical stiffening effect. Conversely, the Epi-on technique was selected for thinner corneas or patients at higher risk of postoperative discomfort, as epithelial preservation reduces pain, infection risk, and epithelial healing time, although it may provide a comparatively weaker cross-linking effect. The inclusion of both techniques reflects real-world clinical decision-making in early-stage keratoconus management.

**Figure 10:**
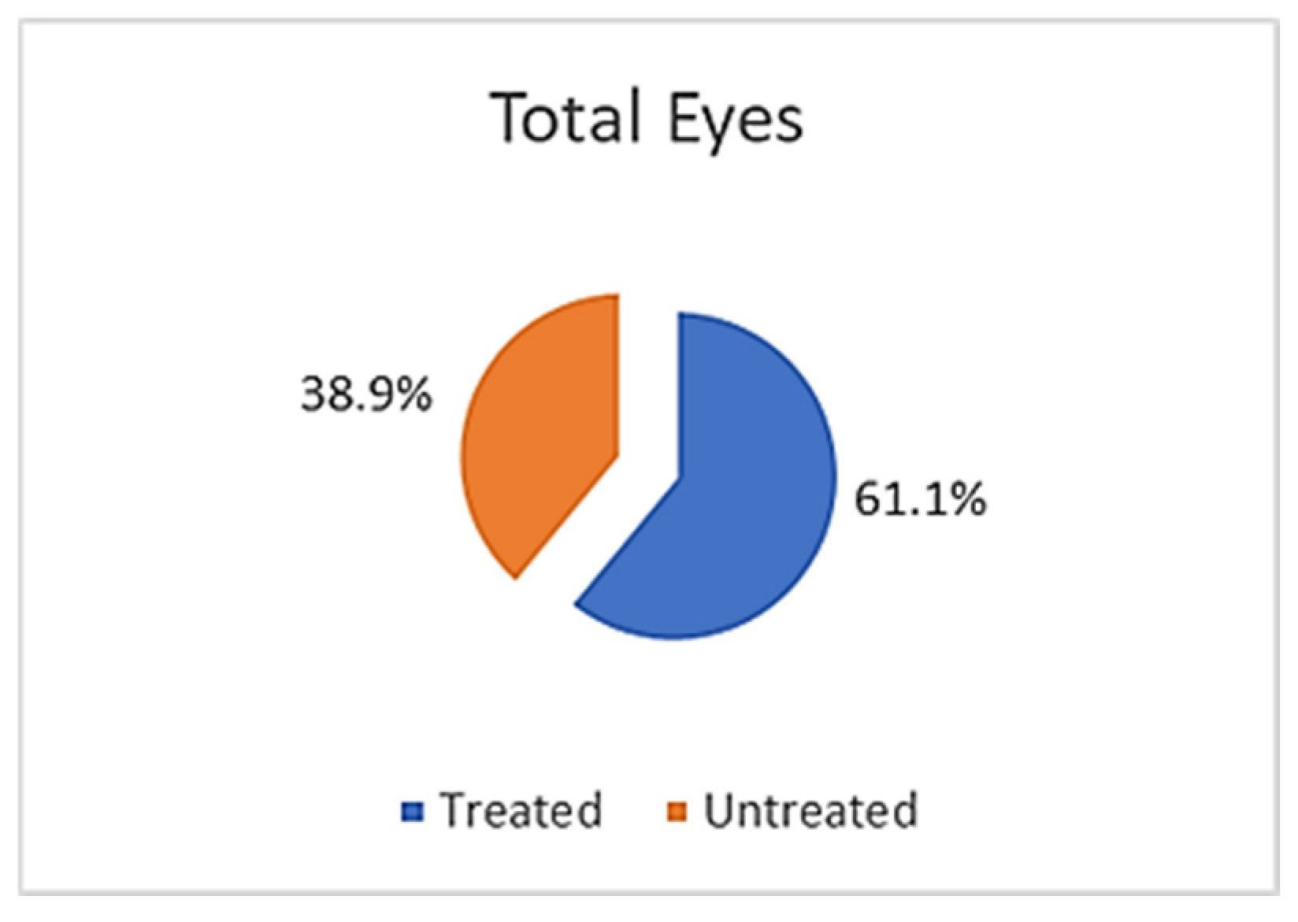
Baseline clinical characteristics of patients with keratoconus (n = number of patients).

**Figure 11:**
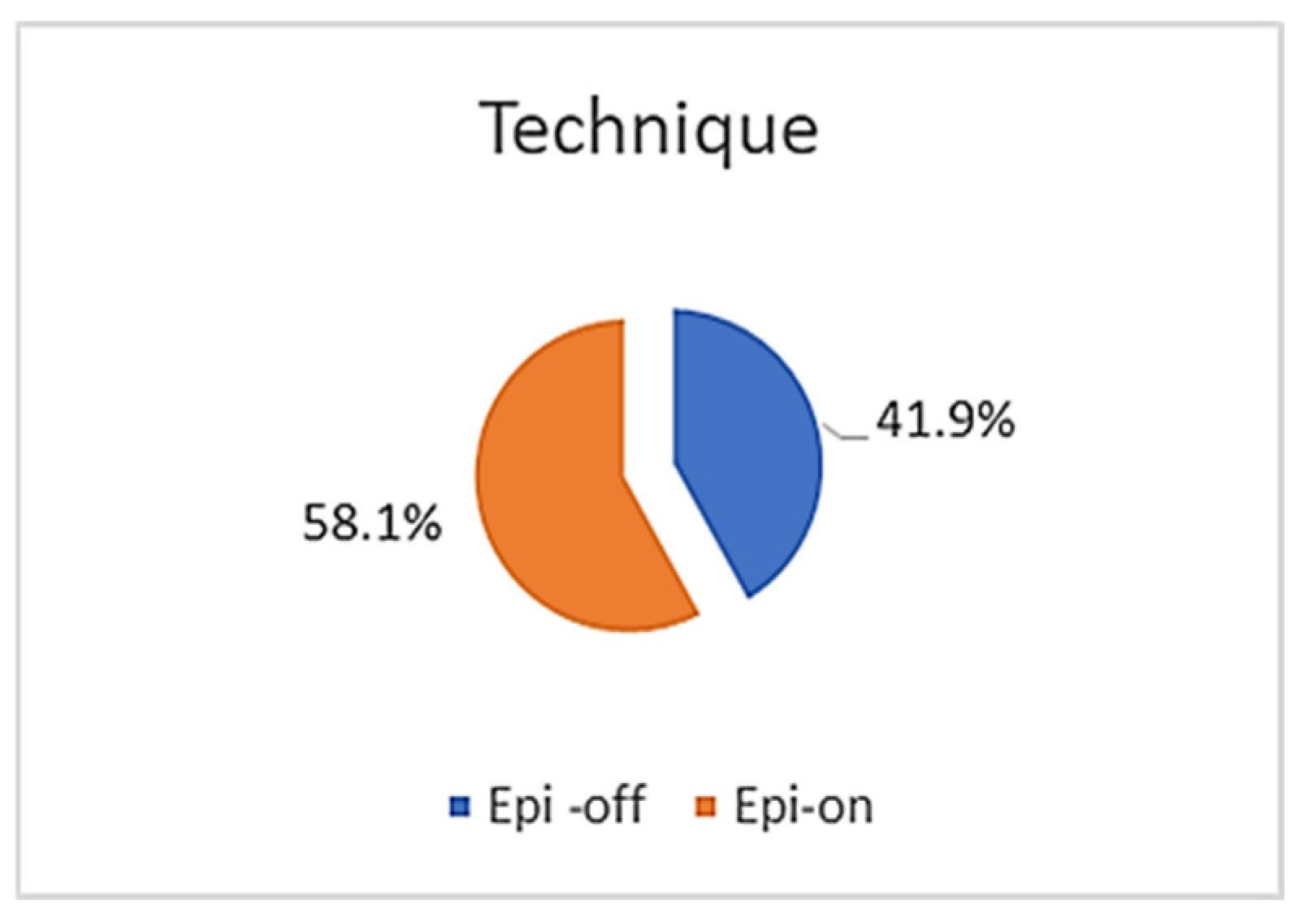
Treatment technique, with the Epi-off and Epi-on techniques (n = number of patients).

The Epi-on approach preserves corneal thickness, reduces postoperative discomfort, and lowers infection risk; however, it may yield less robust outcomes and occasionally requires retreatment (Figure 10 and Figure 11). The Belin ABCD system now provides a more comprehensive classification framework, as a result of recent challenges regarding the diagnosis and treatment of KC [43]. According to the Amsler–Krumeich (AK) classification, our KC data were categorized as follows: early stage, 55.0%; second stage, 24.4%; third stage, 5.7%; and advanced stage, 6.5%. We concluded that the largest number of patients were in the first group (*p* = 0.935) (Table 1). The high prevalence of early stage in our sample suggests that the increasingly widespread use of screening and tomographic analysis enables the detection of disease before loss of function.

**Figure 12:**
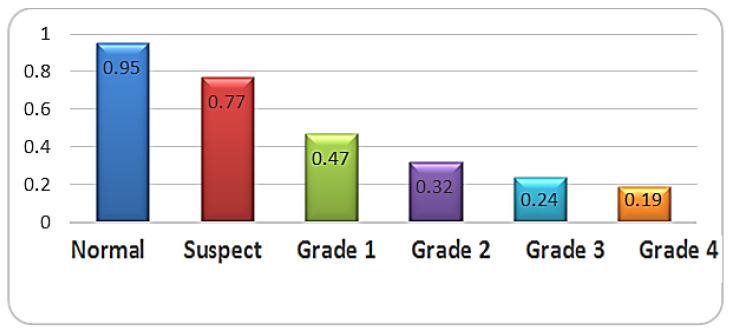
Distribution of BCV values according to study group (n = number of patients).

Because KC affects both eyes but often progresses asymmetrically, patients frequently experience fluctuating dioptric changes and a decline in BCVA that cannot be fully corrected with spectacles or contact lenses. In our cohort, although only 0.8% of eyes were initially classified as suspect KC, tomographic analysis revealed early ectatic features in the fellow eye as well (Figure 12). Parameters such as progression indices, posterior elevation and thickness of the epithelium have a special diagnostic value in subtle forms, as well as that modern devices enable the detection of deviations that are clinically invisible, thereby reducing the risk of late diagnosis in the contralateral eye [43,44].

KC is most often diagnosed at an advanced or progressive stage, where symptoms usually appear in the later stages. However, similar to our research results, early-stage (subclinical) KC was asymptomatic, making diagnosis more challenging, requiring topographical, tomographic, and auto-refractometric assessments. Diagnosis of subclinical KC requires combined evaluation of anterior and posterior corneal surfaces; anterior topography alone is insufficient. Previous studies have reported low rates of suspected KC, likely because most patients present with early-stage disease, as reflected by the 55% of respondents in our study who were classified as grade 1. These findings suggest that the detection of subclinical forms relies on a multimodal approach [3,44,45,46].

Unilateral or bilateral KC was present in all participants. The right eye was unaffected in 6.9% and the left in 8.4%, with no sex-related differences (Table 3). Younger patients (22.4%) were more often in advanced stages (*p* = 0.235), underscoring the need for prompt intervention. Age was not significantly associated with KC presence or severity (Table 1). Although age is often cited as a predictor of progression, numerous authors are of the opinion that individual biomechanical parameters are reliable indicators. Therefore, a comprehensive analysis of structural and functional indicators is recommended, especially in young patients, in order to identify those with a potentially faster course of the disease [3,47,48,49,50,51,52,53,54,55].

Keratometric readings revealed significantly lower K1 and K2 values in normal and suspect eyes compared with higher-grade KC (*p* < 0.001). Kmax values were similarly lowest in normal and suspect eyes. Corneal thickness varied significantly across groups, with grades 3 and 4 showing the greatest thinning (Figure 6). KC progression may occur without concurrent changes in Kmax, as previously documented. Although Kmax is widely used to monitor ectasia, a universal threshold for progression remains undefined. Choi et al. found that K1, K2, and Km changes more reliably distinguish between stable and progressive KC: changes in keratometric parameters reflect the complexity of pathophysiological processes in KC, so optimal monitoring of keratometric and structural indicators is necessary [3,53,54,55]. Early ectatic changes typically arise on the posterior surface before manifesting anteriorly. Given the association between increasing steepening and structural compromise in thin corneas, pachymetry is critical for detecting suspect KC. Our data confirmed significant thinning at grade 4. BCVA declined substantially in advanced KC (*p* < 0.001), although BCVA alone is not a dependable indicator of progression, particularly in patients using corrective lenses. Emerging artificial intelligence technologies may improve early KC detection and future disease management: AI models based on a combination of tomographic, biomechanical and epithelial data already show extremely high potential for early detection and prediction of progression [55,56]. The use of corneal tomography for the assessment of keratometric parameters (K1, K2, Kmax) and pachymetry is supported by previous studies demonstrating their diagnostic value in both subclinical and manifest keratoconus. In particular, changes in posterior corneal elevation and thickness distribution have been shown to precede anterior surface alterations, reinforcing the importance of tomographic evaluation in early disease detection. Recent case-based analyses further support the clinical relevance of these parameters in characterizing early ectatic changes and monitoring disease progression [57]. Although the title emphasizes the impact of early keratoconus screening on quality of life, no validated quality-of-life questionnaires were applied in this study. Therefore, quality of life was assessed inferentially, based on clinical and tomographic indicators known to correlate with visual function, including keratometric stability, corneal thickness preservation, and best-corrected visual acuity. Early stabilization of these parameters through timely intervention is widely recognized as a key factor in maintaining functional vision and minimizing the psychosocial burden associated with keratoconus progression. Future prospective studies incorporating validated keratoconus-specific quality-of-life instruments are needed to directly quantify these patient-reported outcomes.

This study has several limitations. Its retrospective and single-center design may introduce selection bias related to referral patterns. Risk factors such as eye rubbing were self-reported and subject to recall bias. The absence of longitudinal follow-up precluded assessment of true disease progression and long-term treatment outcomes. In addition, quality of life was not directly measured using validated questionnaires. These limitations should be considered when interpreting the findings.

## 5. Conclusions

Patients suffering from KC experience a decline in quality of life regardless of treatment. Disease progression can significantly affect vision; therefore, early screening enables timely treatment (CXL). The evolution of this technique has contributed to preventing and slowing disease progression. The most valuable finding of this study is the high proportion of patients diagnosed at an early stage of keratoconus, highlighting the effectiveness of tomographic screening in detecting disease before significant visual deterioration occurs. Early identification allowed timely implementation of corneal collagen cross-linking, particularly in younger patients at higher risk of progression. These results support the integration of routine corneal tomography into screening protocols for adolescents and young adults with refractive astigmatism, eye rubbing, or a positive family history. From a clinical perspective, structured early screening strategies may reduce the need for invasive interventions such as corneal transplantation and contribute to long-term preservation of visual function.

While early keratoconus screening appears to facilitate timely intervention with corneal collagen cross-linking, these findings should be interpreted with caution due to the retrospective, single-center design and lack of longitudinal progression data. The results primarily reflect cross-sectional detection and treatment patterns. Prospective, longitudinal screening studies incorporating functional and patient-reported outcomes are warranted to better assess the long-term impact of early detection on disease progression and quality of life.

## Figures and Tables

**Table 1:** Mean age stratified by keratoconus grade.

		Age (Year)			
Keratoconus	N	Mean	SD		
Normal	11	20.0	3.8		
Suspect	1	23.0	-		
Grade 1	63	23.3	4.1		
Grade 2	38	24.0	5.6		
Grade 3	8	26.8	6.7		
Grade 4	10	22.4	4.8		
Total	131	23.4	4.9		
*p*-value	*p* = 0.235				

n = number of patients; “values are presented as mean ± SD”.

**Table 2:** Factors linked to keratoconus.

Factor	Relative Risk
Allergy	1.4
Asthma	1.9
Eczema	3.0
Eye rubbing	3.1
Family history	6.4

**Table 3:** Prevalence in both eyes according to gender.

	M		F		Total		*p*-Value
	N	%	N	%	N	%	
Total	78	100.0	53	100.0	131	100.0	
OD							
KCN	71	91.0	50	94.3	121	92.4	*p* = 0.739
Non-KCN	6	7.7	3	5.7	9	6.9	
Suspect	1	1.3	-	-	1	0.8	
OS							
KCN prevalence	71	91.0	48	90.6	119	90.8	*p* = 0.999
Non-KCN prevalence	7	9.0	4	7.5	11	8.4	
Suspected	-	-	1	1.9	1	0.8	

KCN: keratoconic eyes, Non-KCN: non-keratoconic eyes, Suspect: keratoconic eyes, n: number of patients.

## Data Availability

The raw data supporting the conclusions of this article will be made available by the authors on request.

## Abbreviations

The following abbreviations are used in this manuscript:

KC	Keratoconus
VA	Visual acuity
